# Axonal outgrowth, neuropeptides expression and receptors tyrosine kinase phosphorylation in 3D organotypic cultures of adult dorsal root ganglia

**DOI:** 10.1371/journal.pone.0181612

**Published:** 2017-07-24

**Authors:** Estrela Neto, Cecília J. Alves, Luís Leitão, Daniela M. Sousa, Inês S. Alencastre, Francisco Conceição, Meriem Lamghari

**Affiliations:** 1 i3S - Instituto de Investigação e Inovação em Saúde, Universidade do Porto, Porto, Portugal; 2 INEB—Instituto de Engenharia Biomédica, Universidade do Porto, Porto, Portugal; 3 FMUP—Faculdade de Medicina da Universidade do Porto, Porto, Portugal; 4 ICBAS—Instituto de Ciências Biomédicas Abel Salazar, Universidade do Porto, Porto, Portugal; University of California Los Angeles, UNITED STATES

## Abstract

Limited knowledge from mechanistic studies on adult sensory neuronal activity was generated, to some extent, in recapitulated adult *in vivo* 3D microenvironment. To fill this gap there is a real need to better characterize the adult dorsal root ganglia (aDRG) organotypic cultures to make these *in vitro* systems exploitable for different approaches, ranging from basic neurobiology to regenerative therapies, to address the sensory nervous system in adult stage. We conducted a direct *head-to-head* comparison of aDRG and embryonic DRG (eDRG) organotypic culture focusing on axonal growth, neuropeptides expression and receptors tyrosine kinase (RTK) activation associated with neuronal survival, proliferation and differentiation. To identify alterations related to culture conditions, these parameters were also addressed in retrieved aDRG and eDRG and compared with organotypic cultures. Under similar neurotrophic stimulation, aDRG organotypic cultures displayed lower axonal outgrowth rate supported by reduced expression of growth associated protein-43 and high levels of RhoA and glycogen synthase kinase 3 beta mRNA transcripts. In addition, differential alteration in sensory neuropeptides expression, namely calcitonin gene-related peptide and substance P, was detected and was mainly pronounced at gene expression levels. Among 39 different RTK, five receptors from three RTK families were emphasized: tropomyosin receptor kinase A (TrkA), epidermal growth factor receptors (EGFR, ErbB2 and ErbB3) and platelet-derived growth factor receptor (PDGFR). Of note, except for EGFR, the phosphorylation of these receptors was dependent on DRG developmental stage and/or culture condition. In addition, EGFR and PDGFR displayed alterations in their cellular expression pattern in cultured DRG. Overall we provided valuable information particularly important when addressing *in vitro* the molecular mechanisms associated with development, maturation and regeneration of the sensory nervous system.

## Introduction

The understanding of cellular and molecular mechanisms behind neuronal development, regeneration, and targeted innervation of peripheral tissues in physiological and pathological conditions has long been searched by neurobiologists. Although many significant contributions have been obtained, there are still many challenges to be surpassed.

The difficulty to mimic live systems *in vitro* has been an important setback for biochemical research. Particularly in neuroscience field, *in vitro* tools have evolved tremendously to better replicate physiological and pathological conditions. Neurons derived from dorsal root ganglia (DRG) are widely used to conduct studies on the molecular mechanisms controlling many different neuronal functions of the peripheral nervous system. These include studies on neuronal development [1], axonal growth, regeneration and degeneration [2,3], axonal transport [4], myelination [5], targeted innervation [6,7] and platforms for pharmacological drug screening, patch-clamp electrophysiology or calcium imaging [8,9].

There are several types of DRG neuronal cultures, each one exhibiting particular features, advantages and disadvantages. DRG neurons can be collected at different stages of neuronal development, whether they are obtained from embryos, newborn or adult animals. They can also be retrieved from different species, namely rat, mouse or chicken [1,10–14]. Upon dissection, the collected ganglia from embryonic or adult origin can be cultured as dissociated cells or explants.

Explant organotypic cultures are viewed as an attractive option since they are more physiologically relevant than dissociated cells and the original *in vivo* three-dimensional (3D) architecture is preserved. The cell-cell communication among neurons, Schwann cells and resident macrophages is not disrupted, neither is the 3D microenvironment and extracellular matrix organisation. Currently, most studies rely on explant embryonic DRG culture. They are commonly used to dissect the molecular mechanisms of controlling the different neuronal functions of the peripheral nervous system *in vitro*. They have been of key importance to the study of the signaling pathways underlying neuronal regeneration at the molecular level. However, they entail limitations when aiming to infer and design strategies to promote axonal regeneration of mature neurons. Embryonic neurons depend on neurotrophins to survive during the first days in culture [15]. Furthermore, it is accepted that the embryonic cells have a potential to counteract regeneration barriers that adult cells cannot surpass [16–18].

These limitations lead investigators to seek other alternatives. Postnatal and adult DRG sensory neurons offer the possibility to study mature, completely developed cells that may better resemble the *in vivo* features of sensory neurons [15]. However, these studies were mainly addressed in dissociated adult DRG sensory neurons. Currently, few studies employing DRG explants from adult origin were reported [19–22].

This implies that limited *in vitro* mechanistic studies associated with adult sensory neuronal activity in the complex niche involving other relevant cell types, as well as the appropriate spatial arrangement and connectivity, are available. In addition, extrapolation from embryonic setting to the adult situation is hampered. Therefore, there is a real need to better characterize the adult DRG organotypic cultures to make these *in vitro* systems exploitable for different approaches ranging from basic neurobiology to regenerative therapies addressing the sensory nervous system in “adult condition”.

In this study, a comparative study between adult and embryonic DRG organotypic cultures was explored, focusing on axonal outgrowth, neuropeptides expression and tyrosine receptors activation associated with neuronal survival, proliferation and differentiation. To identify alterations associated with the culture conditions, these parameters were also addressed in retrieved adult and embryonic DRG and compared with DRG organotypic cultures.

## Materials and methods

All experiments using animals were carried out with the permission of the local animal ethical committee in accordance with the EU Directive (2010/63/EU) and Portuguese law (DL 113/2013) and approved by the ethics committee of the Portuguese official authority on animal welfare and experimentation (*Direção-Geral de Alimentação e Veterinária*). Adult male mice were sacrificed using carbon dioxide chamber, while pregnant females were sacrificed by cervical dislocation.

### Isolation of dorsal root ganglia (DRG)

#### Retrieved DRG

Embryonic lumbar (L1-L6) DRG were obtained from 16 to 18 days-old (E16-18) C57BL/6 embryos. The embryos were kept in ice-cold Hank’s balanced salt solution (HBSS, Invitrogen) after decapitation. The intact column including the spinal cord, DRG and surrounding cartilaginous tissues were fixed and processed for immunohistochemistry. For protein and RNA extraction, the DRG were separately isolated by cleaning the meninges, cutting the roots off and kept in proper lysis buffer.

Adult lumbar (L1-L6) DRG were obtained from 7-weeks-old C57BL/6 male mice and were retrieved from the excised spine. Briefly, the spine was cleaned of surrounding conjunctive tissue and opened using a scissor. The spinal cord was carefully removed, and the DRG exposed under a stereoscopic magnifier. The meninges were cleaned from the isolated DRG and the roots were cut. DRG were afterwards used for immunohistochemistry, protein and RNA extraction.

#### *In vitro* organotypic cultures

Embryonic ganglia were reached through the dorsal side of the embryo after spinal cord removal. The meninges were cleaned from the isolated DRG and the roots were cut. The ganglia were kept in cold HBSS until seeding. DRG were isolated under the stereoscopic magnifier and seeded into the lower wells of a 15-well μ-Slide Angiogenesis plate from Ibidi (Cat. No. 81506) embedded in the fibrin solution. Fibrin hydrogels were formed by applying equal volumes of a solution of plasminogen-free fibrinogen, pooled from human plasma, and a thrombin solution containing CaCl_2_ and aprotinin (final concentration of fibrin components: 6 mg/mL fibrinogen; 2 NIH U/mL thrombin from human plasma; 2.5 mM CaCl_2_; 10 μg/mL aprotinin). Before being used in the preparation of fibrin gels, fibrinogen was dissolved in ultra-pure water, dialysed against tris-buffered saline (TBS, pH 7.4), sterile-filtered and diluted to 12 mg/mL with sterile TBS. The fibrin gel was allowed to polymerize for 30 min at 37°C in a 5% CO_2_ humidified incubator, before the addition of culture media. DRG were cultured with neurobasal medium supplemented with 2% v/v B-27 Serum-Free Supplement® (B-27, Invitrogen), 60 μM 5-fluoro-2'-deoxyuridine (FDU, Sigma-Aldrich), 25 mM glucose (Glu, Sigma-Aldrich), 1 mM pyruvate (Sigma-Aldrich), 50 ng/ml 7S Nerve Growth Factor (NGF, Calbiochem), 2 mM glutamine (Q, BioWitacker) and 1% penicillin/streptomycin (P/S). Embryonic DRG explant cultures were left undisturbed for 24 h.

Adult ganglia collected as previously mentioned, were kept in cold HBSS until seeding. DRG were isolated under the stereoscopic magnifier and seeded in 96 well-plates embedded in the fibrin solution. Fibrin hydrogels were formed as previously described. DRG were cultured in neurobasal medium as above mentioned. The culture was left undisturbed for 3 days. At this time-point, half of the culture medium was replaced by fresh medium. The culture was again left undisturbed until the 7^th^ day *in vitro*.

Cell viability assay was performed in both embryonic and adult organotypic DRG explant cultures. Briefly live/dead assay was performed for the cell viability assessment, at the end of the established culture period, Explants were incubated with Calcein AM (Invitrogen) in PBS for 30 min at 37°C. Calcein AM was washed out and DRG were incubated with propidium iodide (Sigma Aldrich) for 10 min at 37°C. Images were acquired using IN Cell Analyzer 2000 equipped with IN Cell Investigator software (GE Healthcare, United Kingdom).

### Analysis of calcitonin gene-related peptide (CGRP) and substance P (SP) expression profile

Freshly isolated embryonic and adult DRG were retrieved from the animals and immediately processed for immunohistochemistry (retrieved DRG condition). The explant organotypic DRG cultures were used for immunocytochemistry (*in vitro* condition).

RNA of adult and embryonic DRG was extracted using mirVana™ miRNA isolation kit (Ambion, USA), according to manufacturer’s instructions. The concentration was determined by spectrophotometer (NanoDrop ND1000; NanoDrop Products) and used immediately to proceed with the RT-qPCR. One-step RT-qPCR (Biotool) was used according to manufacturer’s instructions. The primers used for CGRP and SP mRNA amplification can be consulted in S1 Table (S1 Table).

Tissues of retrieved embryonic and adult DRG were collected as previously described, formaldehyde-fixed and embedded in paraffin blocks. For immunohistochemistry, 3-μm thickness cross-sections were deparaffinised and rehydrated before heat-induced antigen retrieval (98°C, 10 mM citrate buffer, pH 6.0). Sections were first incubated with blocking solution 1% BSA (negative control) and then with primary antibodies: anti-CGRP (Sigma-Aldrich) diluted 1:8000 or anti-SP (Millipore) diluted 1:1000 in blocking solution, overnight at 4°C. Afterwards, sections were washed and incubated for 2 h at room temperature (RT) with secondary antibody (Alexafluor 568, Invitrogen). Images were captured with confocal laser scanning microscope (CLSM) Leica SP2 AOBS SE equipped with LCS 2.61 software (Leica Microsystems, Germany).

The adult and embryonic DRG *in vitro* samples were both fixed with 2% paraformaldehyde (PFA, Merk) in phosphate-buffered saline (PBS) during 10 min followed by 10 min at 37°C with 4% of PFA in PBS with 4% sucrose. Ganglia were permeabilized with 0.25% (v/v) Triton X-100 (Sigma-Aldrich) in PBS and incubated, for 30 min at RT, with blocking solution composed of 5% v/v normal goat serum (Invitrogen) and 5% v/v FBS in PBS. DRG were incubated with an antibody directed against neuronal specific marker—βIII tubulin—(Promega) diluted 1:2000, anti-CGRP (Sigma-Aldrich) diluted 1:8000 or anti-SP (Millipore) diluted 1:1000 in blocking solution, overnight at 4°C. Afterwards, cells were washed and incubated 1 h at RT with secondary antibody (Alexafluor 488/568, Invitrogen). Images were captured either with CLSM Leica SP2 AOBS SE or with IN Cell Analyzer 2000 equipped with IN Cell Investigator software (GE Healthcare, United Kingdom).

#### Quantification of neuropeptides expression

CGRP and SP staining intensity was quantified using a custom made software written in MATLAB (TheMathWorks, version 2015a, Natick MA, USA), previously described in [23]. Fixed lower and upper bound values in the intensity levels were used to segment the regions of interest (ROI) by separating background from the foreground and remove eventual labelling artefacts. Mean statistics were calculated for the pixel intensities in the ROIs. The analysis of the retrieved DRG conditions were normalized to the nuclei counting while the *in vitro* condition was normalized to the βIII tubulin positive staining.

### Quantification of axonal growth

**Fig 1:**
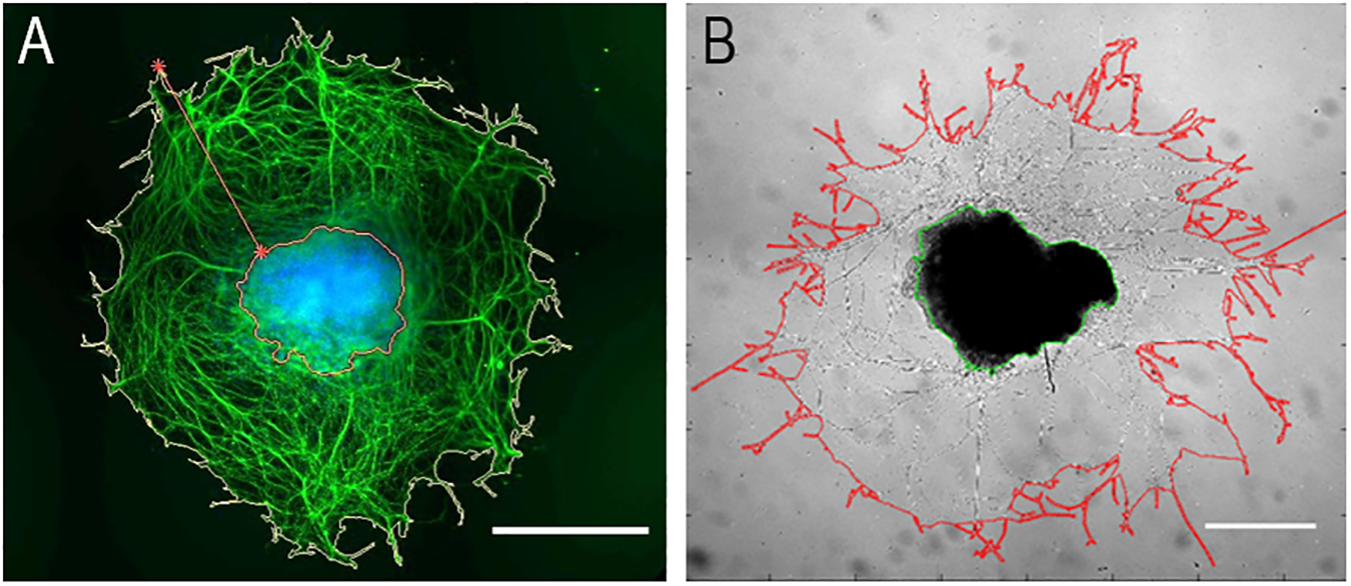
**Axonal growth quantification of embryonic (A) and adult (B) dorsal root ganglion (DRG).** Images were quantified using Bessa *et al*. [23] algorithm developed for quantification of axonal outgrowth. **A.** Fluorescence image of embryonic DRG stained against βIII tubulin (green) and nuclei (blue). Scale bar: 100 μm. **B.** Phase-contrast image of adult DRG. Scale bar: 500μm.

Axonal outgrowth was quantified for an interval of 24 h in culture (from day 1 to day 2, for embryonic DRG (eDRG), and from day 7 to 8 for adult DRG (aDRG)). After 24 h of culture, radial outgrowth was determined. In this study radial outgrowth was defined as the area comprised between the ganglion edge and the outgrowth front. Areas were automatically computed, and the outgrowth area quantified (Fig 1) according to Bessa *et al*. [24]. Moreover, quantification of growth associated protein (GAP-43) and nerve growth factor (NGF) receptor (tropomyosin receptor kinase A, TrkA) were analyzed by RT-qPCR, as previously described in 2.2. The primers used for GAP-43 and TrkA mRNA amplification can be consulted in S1 Table (S1 Table)).

### Analysis of activation and expression of Phospho-Receptor Tyrosine Kinase (RTK)

Proteome profiler mouse phospho-RTK array kit (R&D system, ARY014) was used to quantify the phosphorylation level of 39 tyrosine kinase receptors, following the manufacturer’s instructions. Briefly, the membrane containing immobilised phospho-RTK was initially blocked with BSA at room temperature for 1 h, followed by an overnight incubation with the lysates of embryonic and adult DRG from retrieved and *in vitro* conditions, at 4°C. After a series of washes, the membrane was incubated with a Detection Antibody Cocktail, then with horseradish peroxidase-conjugated secondary antibody (Pierce Chemical, Rockford, IL) and finally with a chemiluminescent detection reagent. Each array membrane was exposed to X-ray film using a chemiluminescence detection system (Amersham). The film was scanned using Molecular Imager GS800 calibrated densitometer (Bio-Rad) and pixel density was presented by quantifying the mean spot densities with Quantity One 1-D Analysis Software, v 4.6 (Bio-Rad).

### Cellular expression pattern of activated RTK

The expression of the most phosphorylated receptors was also analyzed by immunostaining. Retrieved and *in vitro* eDRG and aDRG were processed as previously mentioned in section 2.2. After blocking, DRG were incubated with the following primary antibodies: anti-βIII tubulin and anti-TrkA (Millipore) diluted 1:100, anti- epidermal growth factor receptor (EGFR, Cell Signaling Technology) diluted 1:50 or anti-platelet-derived growth factor receptor alpha (PDGFRα, R&D Systems) diluted 1:250 in blocking solution, overnight at 4°C. Afterwards, samples were washed and incubated for 2 h at room temperature (RT) with respective secondary antibodies. Images were captured with CLSM Leica SP2 AOBS SE.

### Expression analysis of RTK downstream effectors

RNA of adult and embryonic DRG was collected as previously described in section 2.2. Quantification of mRNA expression of RhoA and glycogen synthase kinase 3 beta (Gsk3β) was quantified by RT-qPCR. The primers used for mRNA amplification can be consulted in S1 Table (S1 Table).

### Statistical analysis

Data following a normal distribution are presented as mean ± S.E.M. (standard error of the mean), otherwise they are presented as median ± min/max. Analysis of data was performed using GraphPad Prism 6.00 for Windows (GraphPad Software, San Diego California USA) or R software (version 3.3.2) [25]. Detailed information on statistical analysis can be found in figures caption. Differences between groups were considered statistically significant when p<0.05 (* p<0.05; ** p<0.01; *** p<0.001).

## Results

### CGRP and SP neuropeptides expression profiles

CGRP and SP, neuropeptide mediators of sensory nervous system function, are the two most important neuropeptides expressed by sensory nerve fibers. CGRP and SP mRNA and protein expression were analyzed in adult and embryonic DRG explants cultures. Comparison with expression levels in retrieved DRG was also performed.

**Fig 2:**
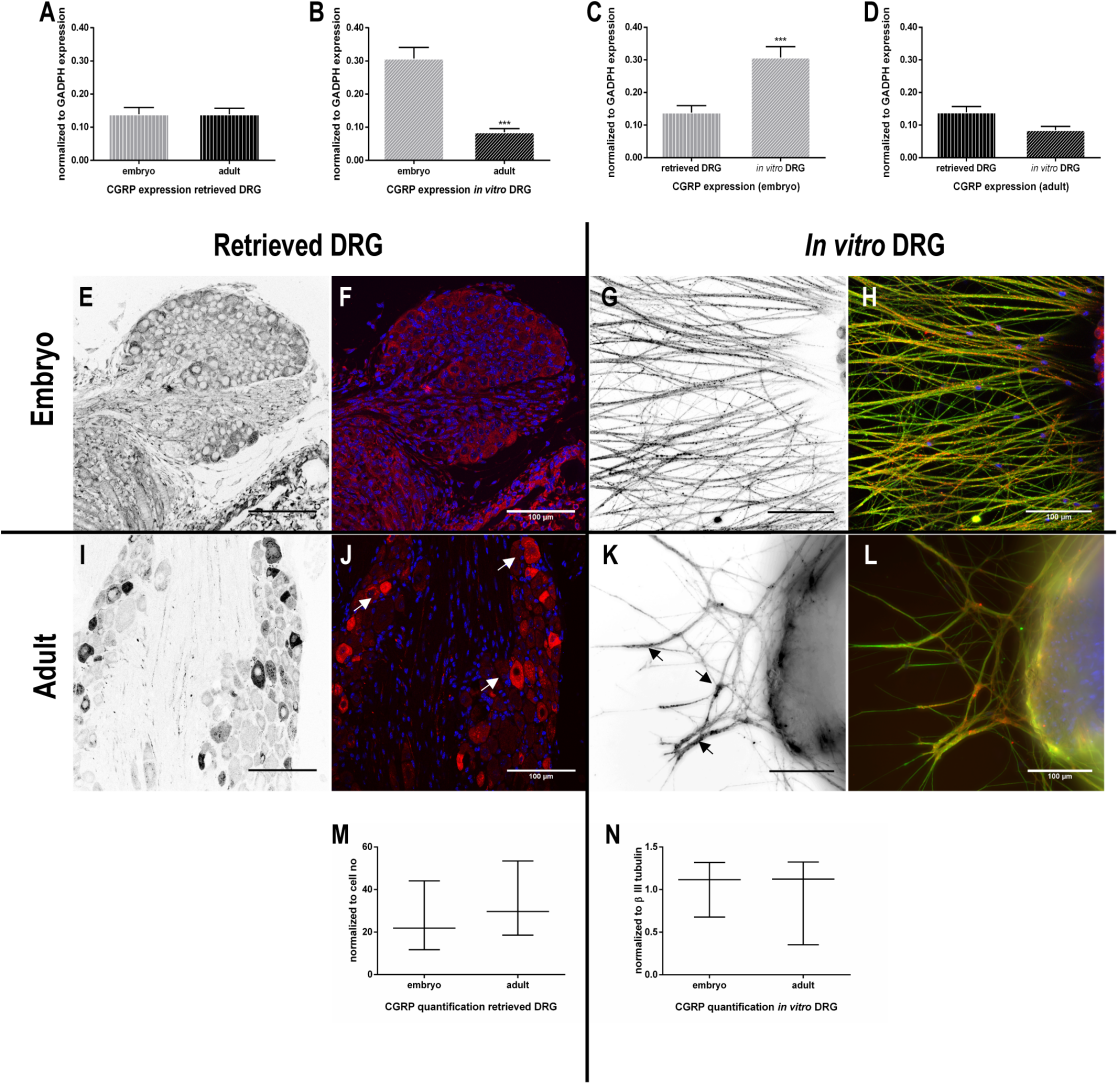
Expression of calcitonin gene-related peptide (CGRP) in DRG. RT-qPCR analysis of CGRP expression by retrieved and *in vitro* embryonic and adult DRG (A-D) (n = 3–8). Data are represented as mean ± SEM (standard error of the mean). The normality of the residuals (value-mean) in the linear regression of the independent variables was tested. Afterwards, a two-way ANOVA was performed followed by Tukey’s post-hoc multiple comparison test (retrieved and *in vitro* / eDRG and aDRG). Representative images of CGRP immunostaining (E-L) are depicted. Images in black and white are related to the channel corresponding to the immunostaining of CGRP alone (red channel on merged images). βIII tubulin is stained in green for the *in vitro* conditions and nuclei in blue (G, H, K, L). Scale bar: 100 μm. Quantification of CGRP staining in retrieved DRG, normalized to cell number (M) and normalized to βIII tubulin staining (N) (n = 6–8). Data represented as median ± min/max. Mann-Whitney U test was performed to evaluate the statistical differences (* p<0.05; ** p<0.01; *** p<0.001).

In retrieved DRG, RT-qPCR analyses showed no significant differences in the mRNA expression levels of CGRP between adult or embryonic DRG (Fig 2A). However, immunohistochemistry revealed a different pattern of CGRP expression. In retrieved aDRG higher intensity of immunohistochemical reaction to anti-CGRP was observed in a limited number of neurons (Fig 2I and 2J-white arrows), while in eDRG, CGRP-positive cells showed widespread distribution but with low immunoreactivity (Fig 2E and 2F). The quantification of CGRP positive labelled cells showed a trend towards higher CGRP expression levels in retrieved aDRG than eDRG (Fig 2M).

Under culture conditions, the CGRP mRNA levels in aDRG explants were significantly lower when compared to eDRG (Fig 2B). Comparison between retrieved DRG and DRG explants cultures showed alterations in the CGRP mRNA expression profiles. *In vitro*, a reduction of CGRP mRNA expression occurs in aDRG explants while in the eDRG explants an upregulation of CGRP is observed when compared to retrieved ones (Fig 2C and 2D).

The immunocytochemistry analysis of CGRP axonal expression in DRG explants showed few neurites in aDRG with high-intensity labelling (Fig 2K- black arrows, 2L) whereas in eDRG the majority of fibers were immunoreactive to CGRP (Fig 2G and 2H). However, when quantified, CGRP axonal expression revealed no significant differences between aDRG and eDRG explant cultures (Fig 2N). It is important to remind that these quantifications were normalized to the neurite number to rule out possible bias due to the differences in neurite network density observed between adult and embryonic DRG.

**Fig 3:**
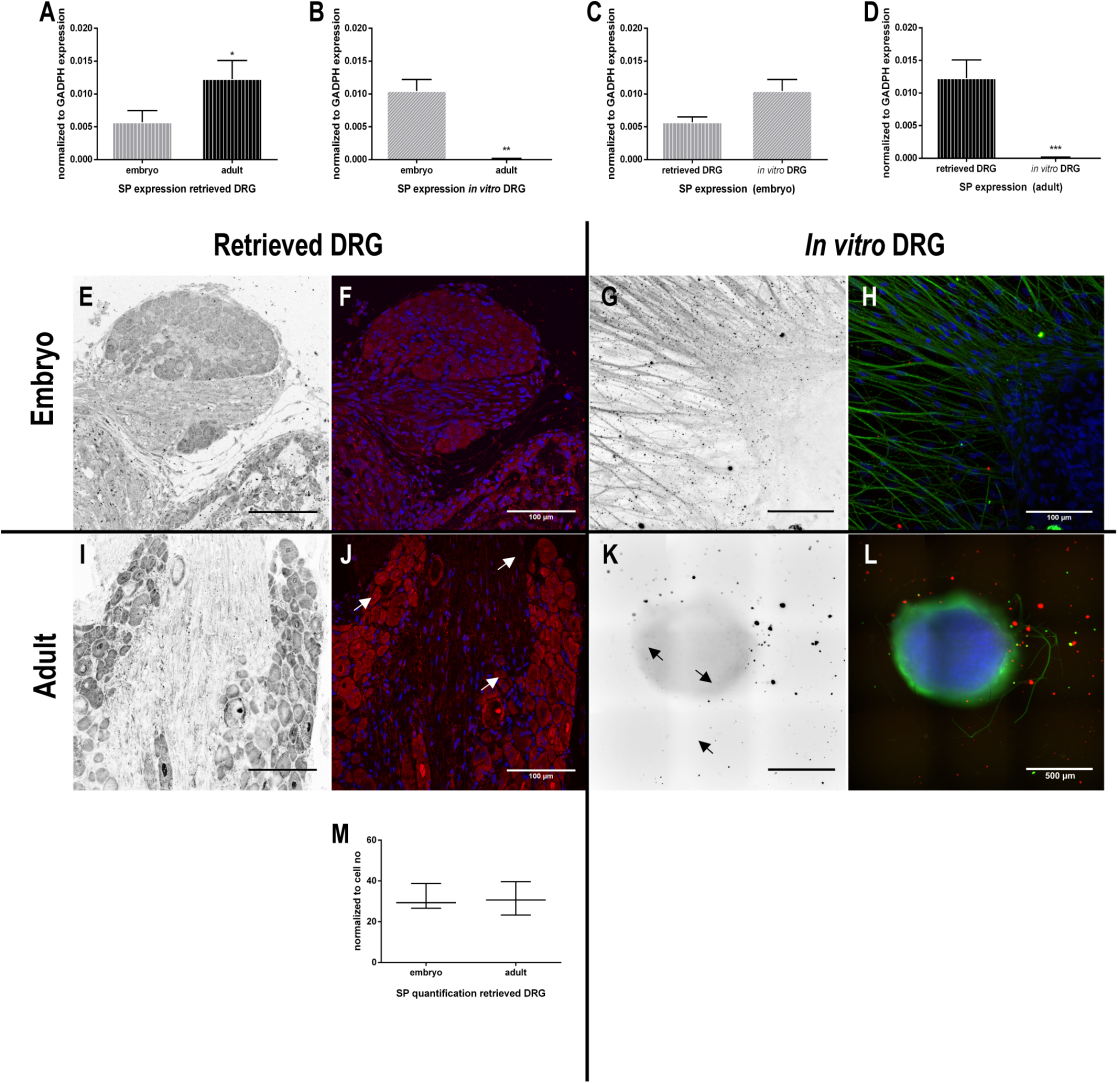
Expression of substance P (SP) in DRG. RT-qPCR analysis of SP expression by retrieved and *in vitro* embryonic and adult DRG (A-D) (n = 3–8). Data are represented as mean ± SEM. The normality of the residuals (value-mean) in the linear regression of the independent variables was tested. Afterwards, a two-way ANOVA was performed followed by Tukey’s post-hoc multiple comparison test (retrieved and *in vitro* / eDRG and aDRG). Representative images of SP immunostaining (E-L) are depicted. Images in black and white are related to the channel corresponding to the immunostaining of SP alone (red channel on merged images). βIII tubulin is stained in green for the *in vitro* conditions and nuclei in blue (G, H, K, L). Scale bar: 100 μm (E-J); 500 μm (K, L). Quantification of SP staining in retrieved DRG, normalized to cell number (M) (n = 4). Data represented as median ± min/max. Mann-Whitney U test was performed to evaluate the statistical differences (* p<0.05; ** p<0.01; *** p<0.001).

Regarding SP mRNA expression in retrieved DRG, aDRG expression levels were significantly higher than eDRG (Fig 3A). These results were confirmed by immunohistochemistry showing a higher intensity in SP-positive neurons in aDRG when compared to eDRG (Fig 3E, 3F, 3I and 3J). When normalized to the cell number, the quantification of the positively labelled cells did not show statistical differences between eDRG and aDRG (Fig 3M). Remarkably, besides the mRNA expression of SP in *in vitro* eDRG (Fig 3D), the protein expression was completely abolished when DRG explants were cultured *in vitro*, both embryonic and adult (Fig 3G, 3H, 3K and 3L).

### Axonal outgrowth

#### Quantification of axonal outgrowth

**Fig 4:**
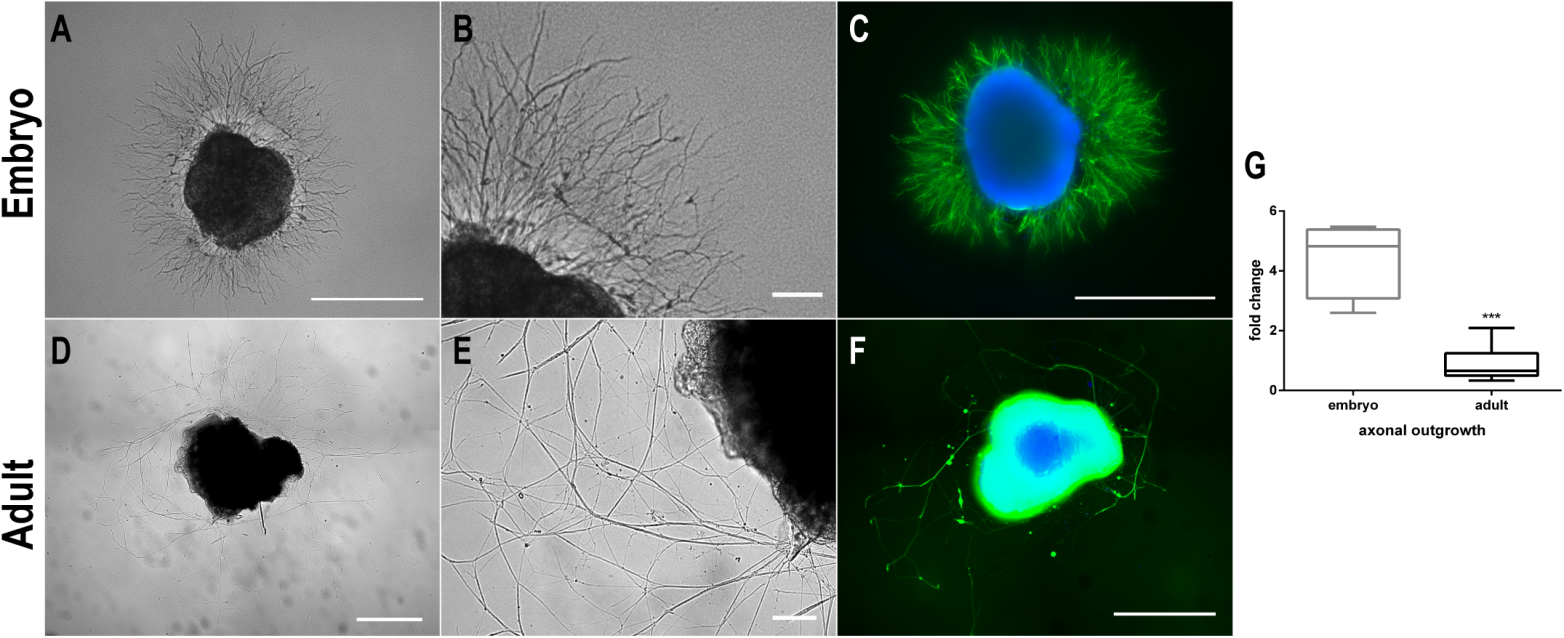
Axonal outgrowth measures of explant organotypic cultures of embryonic and adult DRG. Representative images of embryonic DRG (E17, DIV2) *in vitro* cultures in phase contrast (A, zoom in B) and staining against βIII tubulin (green) and nuclei (blue) (C). Representative images of adult DRG (7 weeks-old, DIV8) *in vitro* cultures in phase contrast (D, zoom in E) and staining against βIII tubulin (green) and nuclei (blue) (F). The graph shows the quantification of axonal outgrowth for the last 24 h in culture (from day 1 to day 2 for embryonic DRG and from day 7 to 8 for adult DRG) (G). Scale bar: 500 μm (A, C, D, F); 100 μm (B, E) (n = 4–16). Data represented as box and whisker plot. Mann-Whitney U test was performed to evaluate the statistical differences (* p<0.05; ** p<0.01; *** p<0.001).

Images of embryonic (A-C) and adult (D-F) DRG explant cultures are displayed in Fig 4. In the first 24 h upon plating, a large sprouting of axons emerging from eDRG explants was observed. This neurite network was dense and homogeneously distributed around the ganglion (Fig 4A). On the other hand, aDRG explants required a longer period to start to project neurites and to fully establish its network (Fig 4D). Viabilty assay performed in these explant cultures, showed few dead cells within the ganglia (S1 Fig). Seven days of *in vitro* culture was the time-point established to end the culture, as previously described [19,20].

Immunostaining against βIII tubulin, a neuronal marker, was also performed and clearly demonstrate that eDRG can develop a higher neurite network *in vitro* when compared to aDRG (Fig 4C and 4F). We also evaluated the potential for neurite projection in a 24 h interval. Our results show a 4-fold increase in the neurite outgrowth area within this 24 h-period for eDRG explants, whereas no increase was verified for aDRG (Fig 4G).

#### GAP-43 and TrkA receptor expression

The expression of GAP-43 is considered to be widely involved in neuronal mechanisms underlying axonal growth, regeneration, and synaptic plasticity [26]. In addition, previous reports indicated that adult sensory neurons exposed to NGF *in vitro* display an upregulation of GAP-43 [20].

**Fig 5:**
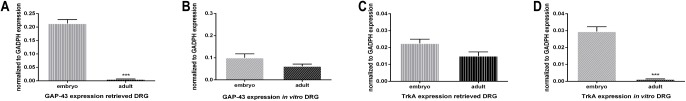
Expression of growth-associated protein 43 (GAP-43) and tropomyosin receptor kinase A (TrkA). Quantification by RT- qPCR of GAP-43 (A, B) and TrkA (C, D) mRNA expression in retrieved and *in vitro* embryonic and adult DRG (n = 4–10). Data represented as mean ± SEM. Two-way ANOVA was performed followed by Tukey’s post-hoc multiple comparison test (* p<0.05; ** p<0.01; *** p<0.001).

In our *in vitro* conditions, both eDRG and aDRG explants were kept in culture under neurotrophic stimulation in the presence of NGF (50 ng/ml). Thus, the levels of GAP-43 and NGF high affinity receptor, TrkA, mRNA transcripts were evaluated by RT-qPCR. The mRNA expression of GAP-43 was significantly lower in the aDRG when compared to the eDRG, both in the retrieved and *in vitro* DRG (Fig 5A and 5B). *In vitro*, aDRG displayed a significant reduction of TrkA mRNA levels when compared to eDRG (Fig 5D). While no significant differences were observed in TrkA receptors mRNA expression in retrieved aDRG and eDRG (Fig 5C).

### Activation profile of receptor tyrosine kinases (RTK)

**Fig 6:**
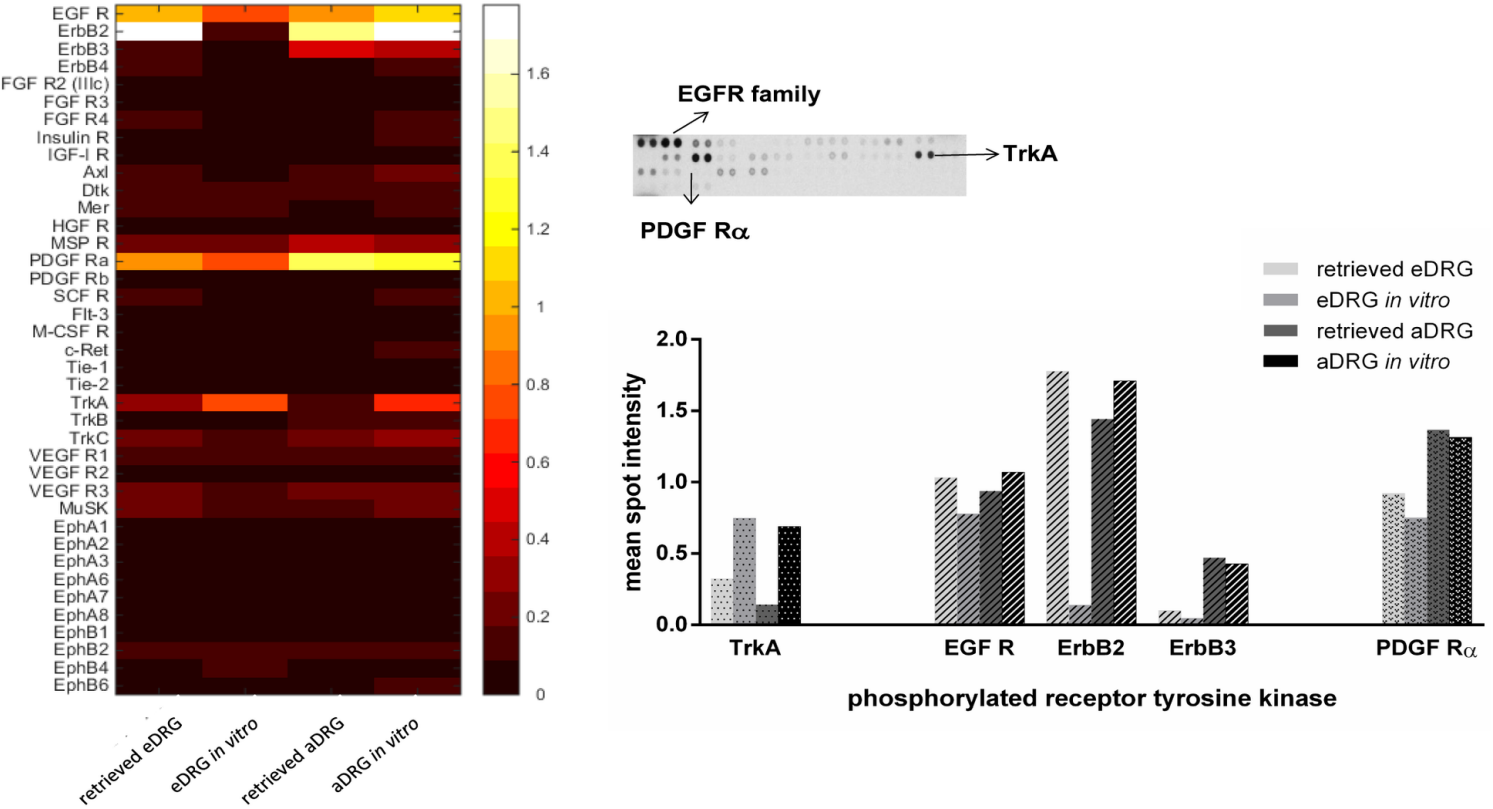
Activation of receptor tyrosine kinases (RTK). Heatmap representing the relative spot intensity for the 39 activated receptors and the four DRG conditions quantified from the X-ray films and representative image of the X-ray film, highlighting the most phosphorylated receptors. The graphic represents the mean spot intensity of the most phosphorylated receptors in retrieved and *in vitro* embryonic and adult DRG, showing the three different families: epidermal-growth factor receptors (EGFR), platelet-derived growth factor (PDGF) receptor alpha and tropomyosin receptor kinase A (TrkA) receptor. For the array analysis, the protein lysate from a pull of 6–10 DRG for each condition (eDRG and aDRG; retrieved and *in vitro*), from three independent experiments (n = 3), was collected. One array was used per condition.

To screen the activation of RTK implicated in neuronal development, growth, survival and axonal regeneration, we assessed the relative level of tyrosine phosphorylation using an antibody array system. This array allows the simultaneous detection of 39 different phosphorylated RTK. The heatmap is a representation of the relative spot intensity, obtained from the quantification of the membranes from the array (Fig 6).

Among the phosphorylated receptors studied, quantitative analysis revealed a higher phosphorylation rate for five tyrosine kinase receptors from different families, namely the tropomyosin receptor kinase (Trk), epidermal growth factor (EGF) receptors, and platelet-derived growth factor (PDGF) receptors (Fig 6).

In accordance with the results obtained for gene expression, TrkA phosphorylation levels of retrieved ganglia were higher in eDRG when compared to aDRG. However, when explants were placed under culture conditions, the phosphorylation levels of TrkA increased in both eDRG (2-fold increase) and aDRG (5-fold increase), reaching similar levels (Fig 6).

Among the EGF receptors family, three different receptors were activated of: EGFR, ErbB2, and ErbB3. Under *in vitro* conditions, the phosphorylation levels of EGFR, ErbB2, and ErbB3 were higher in aDRG explants when compared to eDRG explants. This increase was more pronounced for ErbB2, and ErbB3. Interestingly, except for ErbB2, these differences were not associated to the culture conditions. In fact, in both eDRG and aDRG, EGFR and ErbB3 phosphorylation levels in retrieved DRG were comparable to those obtained *in vitro*. However, ErbB2 phosphorylation suffered a major decrease in eDRG when plated in culture. This does not occur in aDRG (Fig 6).

PDGFRα displayed higher phosphorylation levels *in vitro* in aDRG when compared to those obtained in eDRG, at the same conditions. However, as observed for EGFR and ErbB3, this difference was not an alteration related to the culture condition since no major changes PDGFRα phosphorylation levels were detected when comparing retrieved DRG (embryonic or adult) and DRG (embryonic or adult) under *in vitro* conditions (Fig 6).

### Cellular expression pattern of activated RTK

**Fig 7:**
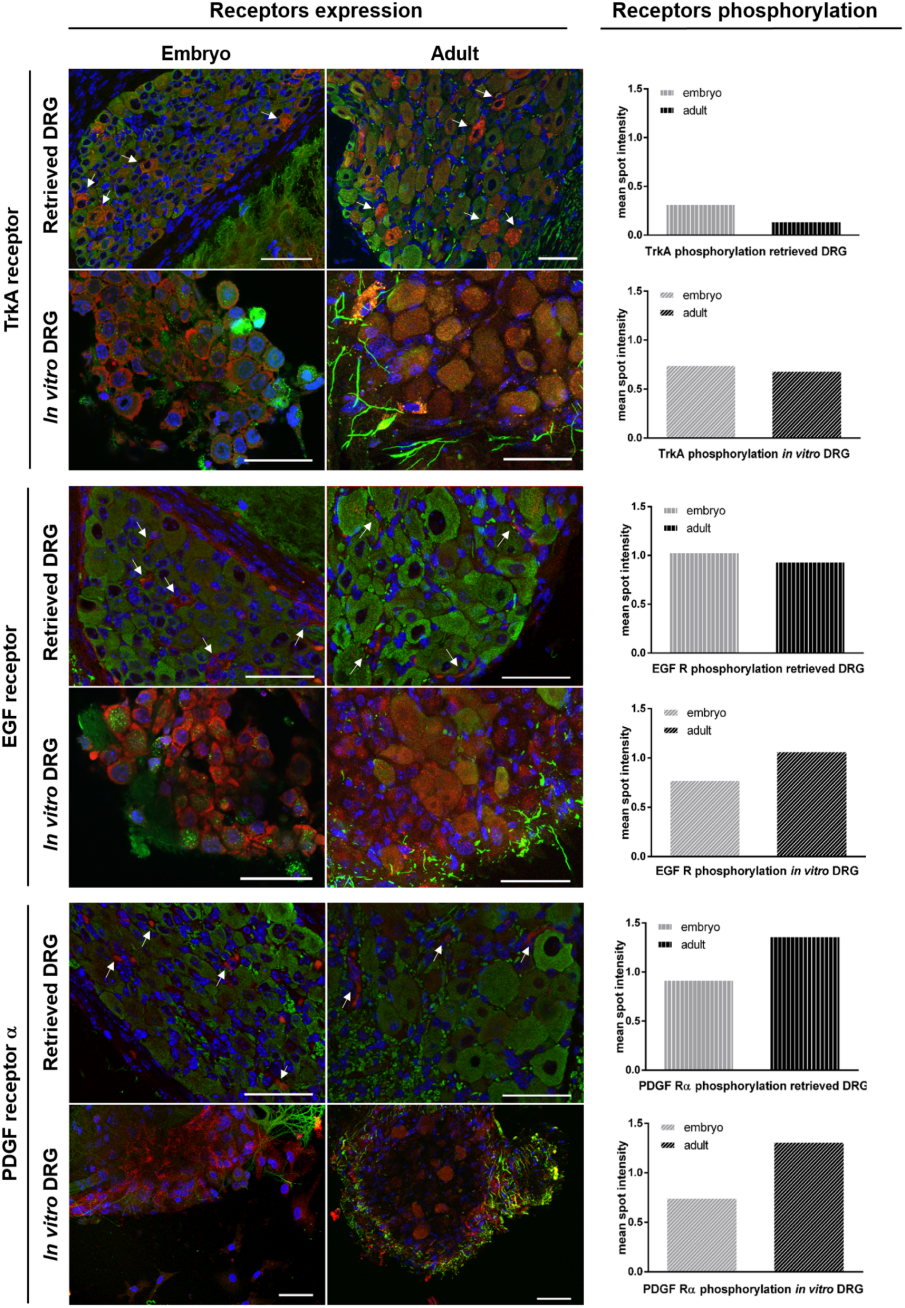
Cellular expression of activated RTK in retrieved and *in vitro* embryonic and adult DRG. Immunostaining of receptor tyrosine kinase (TrkA, EGFR and PDGFRα) in red, βIII tubulin in green and nuclei in blue. Left side images show the cellular pattern of receptors expression (n = 3) in eDRG and aDRG (retrieved and *in vitro* conditions). Right column show the phosphorylation level, in retrieved and *in vitro*, embryonic and adult DRG for each receptor (previously presented in detail in Fig 6).

To analyse the cellular and spatial distribution of the activated receptors within the analyzed DRG, immunostaining was performed on both eDRG and aDRG explants *in vitro* and compared to retrieved DRG. In eDRG and aDRG under culture conditions, TrkA receptor is expressed by cells immunoreactive to βIII-tubulin indicating receptor expression by neurons. A similar pattern was observed in retrieved explants (Fig 7). EGFR and PDGFRα displayed alterations in their expression profiles. In eDRG and aDRG explants cultures, both EGFR and PDGFRα were expressed by glial cells as well as by neurons. However, in retrieved eDRG and aDRG, the expression of both receptors was confined to glial cells (Fig 7).

### Downstream activation of signaling pathways

RTK can activate multiple Rho GTPases. It has been described that the activation of several RTK leads to the rearrangement of the actin cytoskeleton, which is likely mediated through activation of RhoGTPases [27]. To understand the downstream signaling pathway upon activation of the previously analyzed RTK, the expression profile of RhoA and Gsk3β were evaluated by RT-qPCR.

**Fig 8:**
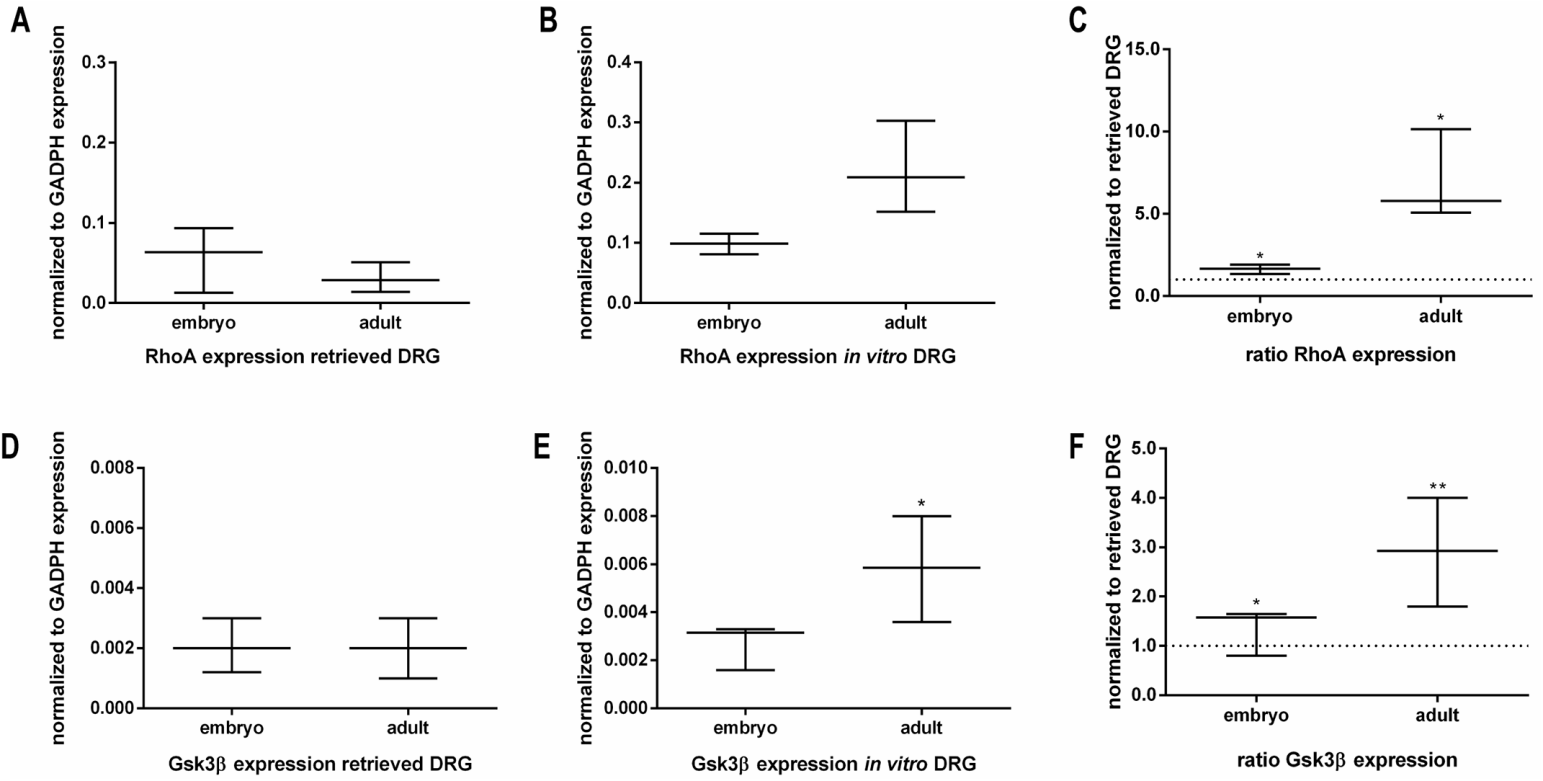
Expression of RhoA and glycogen synthase kinase 3 beta (Gsk3β). Quantification by RT-qPCR of mRNA expression of RhoA by retrieved and *in vitro* embryonic and adult DRG (n = 3–6). The ratio of RhoA expression is the amount expressed *in vitro* normalized to the corresponding retrieved condition (A-C). Quantification by RT-qPCR of Gsk3β expression by retrieved and *in vitro* embryonic and adult DRG (n = 4–6). The ratio of Gsk3β expression is the amount expressed *in vitro* normalized to the corresponding retrieved condition (D-F). Data represented as median ± min/max. Mann-Whitney U test was performed to evaluate the statistical differences (* p<0.05; ** p<0.01; *** p<0.001).

The gene expression analysis showed that under culture conditions aDRG explants express higher RhoA mRNA levels than eDRG, while in retrieved DRG no significative differences were observed (Fig 8A–8C). Downstream of the signaling pathway, the gene expression of Gsk3β also presented similar mRNA levels in retrieved eDRG and aDRG (Fig 8D). However, under culture conditions aDRG explants showed significantly higher Gsk3β expression in comparison to eDRG explants (Fig 8E). In addition, both eDRG and aDRG expressed a higher amount of Gsk3β *in vitro* than the retrieved ganglia (Fig 8F).

## Discussion

In adult DRG organotypic cultures, reduced levels of mRNA encoding for CGRP and SP were detected, when compared to the freshly retrieved explants. Several studies reported the reduction of SP or CGRP synthesis in response to peripheral nerve lesions [28–34]. This decrease can be reversed *in vivo* or *in vitro* by NGF, known to enhance CGRP and SP expression in DRG [35]. In our organotypic systems, DRG explants were kept in culture in the presence of neurotrophic stimulation by NGF (50 ng/ml). Under these conditions, adult and embryonic DRG displayed different CGRP and SP mRNA expression profiles. The NGF stimulatory effect on CGRP and SP mRNA expression was verified in embryonic DRG explants in culture but in adult DRG this effect was not observed. This finding is important to consider in studies addressing the mechanisms of transcriptional regulation of CGRP and SP gene expression in adult DRG *in vitro*. The analysis of neuropeptides immunoreactivity in adult DRG organotypic cultures showed similar CGRP protein axonal content compared to those that were recorded in embryonic DRG organotypic cultures. This suggests that adult DRG organotypic cultures are suitable *in vitro* system where CGRP expression and release, in response to a given excitatory stimulus, can be addressed in “3D adult microenvironment” integrating key factor, such as extracellular matrix and cell-cell interplay between different cell types.

Data on SP protein expression and release were mostly described in dissociated sensory neurons under stimulation of NGF at different concentrations. As an example, it has been reported that dissociated sensory neurons from new born rat express and release SP *in vitro* [36], when exposed to NGF at a concentration of 100 ng/ml. In another report, dissociated mature rat dorsal root ganglion neurons express SP in the presence of 10 ng/ml or 50 ng/ml NGF [37]. In our study, SP protein expression was analyzed in the DRG explants exposed to NGF at a concentration of 50 ng/ml. SP immunostaining revealed an unexpected total lack of protein expression in both adult and embryonic DRG organotypic cultures. We believe that this negative response might not be associated with limited sensitivity of the SP antibody, since it stains SP in retrieved explant, but rather a matter of NGF concentration (50ng/ml) that was not effective in triggering SP protein expression in DRG explants with complex cellular architecture.

A large number of studies showed that embryonic sensory axons grow with greater ability than adult sensory neurons, cultured either as dissociated cells or as explants. Here we conducted a direct *head-to-head* comparison of adult and embryonic DRG axonal growth potency in cultures, under NGF trophic stimulation. Our data showed that adult DRG required longer time to establish neurite network and displayed lower axonal growth rate in a 24 h-period, when compared to embryonic DRG. The expression of GAP-43 is considered to be widely involved in neuronal mechanisms underlying axonal growth and regeneration [26]. In addition, previous reports indicated that adult sensory neurons exposed to NGF *in vitro*, display an upregulation of GAP-43 [20]. Our results are in agreement with the latter observation. GAP-43 gene expression increased 10-fold in adult DRG *in vitro*, when compared to the retrieved explants. Albeit this upregulation, the GAP-43 mRNA levels in adult DRG in culture remained significantly lower than those obtained in embryonic DRG organotypic cultures.

The axonal outgrowth disparity between adult and embryonic DRG cultures was also accompanied by differential expression of TrkA mRNA transcripts. Under the same *in vitro* conditions, adult DRG expresses very low levels of TrkA in comparison to embryonic DRG. Nonetheless, the receptor phosphorylation levels were similar.

Beside TrkA receptor, other classes of receptor tyrosine kinases (RTK) were suggested to be involved in signaling pathways associated with neuronal survival and axon regeneration [38]. To obtain a large overview of the profiling changes in RTK phosphorylation in DRG with different axonal growth capacity, we performed antibody arrays in embryonic and adult DRG organotypic cultures as well as in freshly retrieved explants. Among 39 different receptors, five receptors from three RTK families were emphasised: TrkA, EGFR, ErbB2, ErbB3 and PDGFR alpha. In addition our data demonstrated that, except for EGFR, the phosphorylation level of these receptors is dependent on both the DRG developmental stage and/or the culture conditions.

It has been shown that EGFR inhibitors promoted significant regeneration of injured optic nerves since EGFR blocks the activity of both myelin inhibitors and chondroitin sulphate proteoglycans in inhibiting neurite outgrowth [36]. We observed no major alteration of EGFR phosphorylation between our experimental settings. EGFR phosphorylation levels in adult and embryonic DRG explants, with different rate of axonal outgrowth, were comparable in organotypic cultures. This might suggest that the receptor activation is not directly related to this process. However, a major alteration occurred in the EGFR cellular expression pattern in DRG. In retrieved DRG (adult and embryonic) positive staining for EGF receptor was detected exclusively in glial cells, whereas *in vitro* a spread expression was observed both in glial cells and neurons. This might be related to the injury inflicted during the DRG dissection. This observation is supported by previous studies showing, in response to nerve lesion, a marked upregulation of EGF receptor on non-neuronal and medium-sized neurons [39–41]. PDGFRα has been described to induce anti-apoptotic response leading to survival of neurons upon damage [42]. As described above for EGFR, PDGFRα showed alterations in the cellular expression pattern in DRG organotypic cultures (both adult and embryonic). Its expression was also extended to neuronal cells. This variation is concomitant with higher receptor phosphorylation level that is dependent on the DRG maturation stage.

RTK can activate multiple Rho GTPases. Extensive studies using embryonic *in vitro* culture experiments have shown that activation of RhoA induces growth cone collapse and axonal repulsion [13,43,44]. Our data demonstrated that the levels of RhoA and Gsk-3β mRNA transcripts are higher in adult DRG culture when compared with embryonic DRG culture. This difference is in accordance with a lower axonal growth rate as observed in adult DRG culture.

Overall, herein we provided a valuable insight on the major similitudes and differences occurring in adult or embryonic DRG upon *in vitro* cultures. The delivered information is particularly important when addressing *in vitro* the molecular mechanisms associated with development, maturation, survival and regeneration of the sensory nervous system.

## Supporting information

S1 Fig Cell viability assay. Live/dead assay was performed for the cell viability assessment, at the end of the established culture period, for the organotypic explant cultures of embryonic DRG (A) and adult DRG (B). Explants were incubated with Calcein AM (Invitrogen) in PBS for 30 min at 37°C. Calcein AM was washed out and DRG were incubated with propidium iodide (Sigma Aldrich) for 10 min at 37°C. Images were acquired using IN Cell Analyzer 2000 equipped with IN Cell Investigator software (GE Healthcare, United Kingdom). Scale bar: 100 αm.
(TIF)

S1 File Raw data. (XLSX)

S1 Table Table of primers sequence. (DOCX)
