# Supplementary figures and images for: Axonal outgrowth, neuropeptides expression and receptors tyrosine kinase phosphorylation in 3D organotypic cultures of adult dorsal root ganglia
Source: PLoS One. 2017 Jul 24;12(7):e0181612. doi: 10.1371/journal.pone.0181612 (PMC5524368; doi:10.1371/journal.pone.0181612)

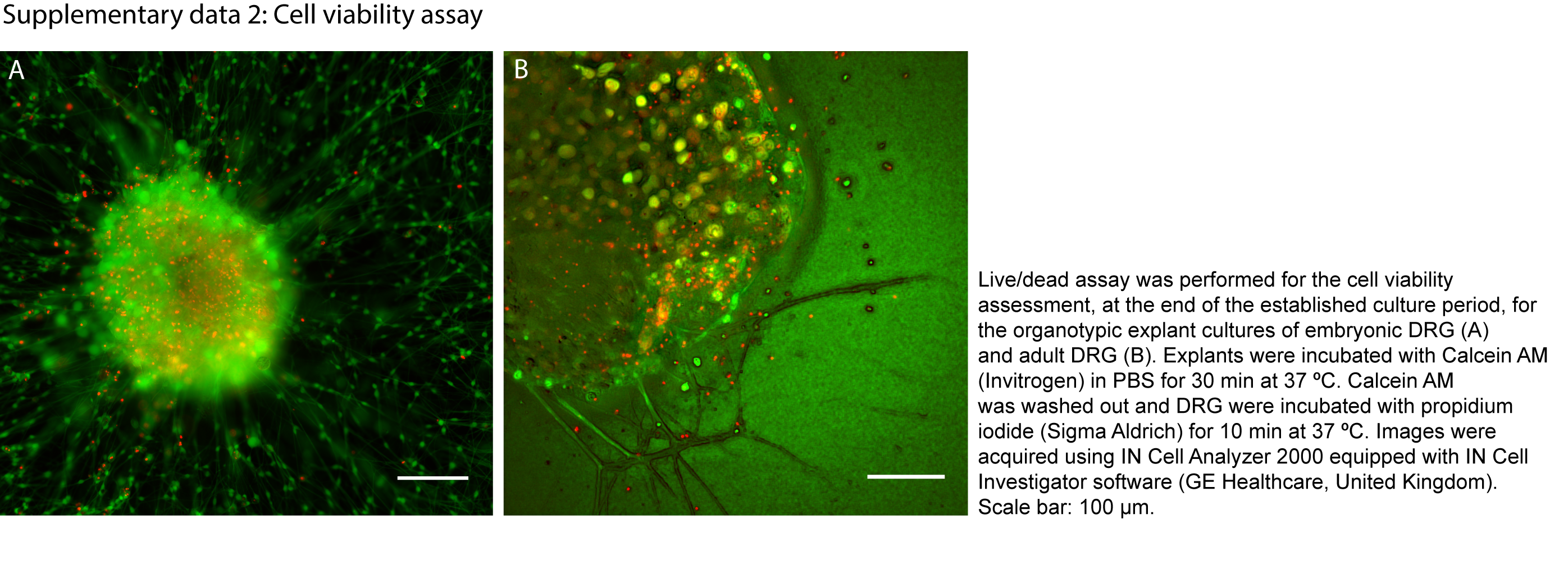

Supplement: S1 Fig — Live/dead assay was performed for the cell viability assessment, at the end of the established culture period, for the organotypic explant cultures of embryonic DRG (A) and adult DRG (B). Explants were incubated with Calcein AM (Invitrogen) in PBS for 30 min at 37°C. Calcein AM was washed out and DRG were incubated with propidium iodide (Sigma Aldrich) for 10 min at 37°C. Images were acquired using IN Cell Analyzer 2000 equipped with IN Cell Investigator software (GE Healthcare, United Kingdom). Scale bar: 100 αm. (TIF) [file pone.0181612.s001.tif]
