# Supplementary material for: Axonal outgrowth, neuropeptides expression and receptors tyrosine kinase phosphorylation in 3D organotypic cultures of adult dorsal root ganglia
Source: PLoS One. 2017 Jul 24;12(7):e0181612. doi: 10.1371/journal.pone.0181612 (PMC5524368; doi:10.1371/journal.pone.0181612)
Supplement: S1 Table — (DOCX) [file pone.0181612.s003.docx]

**Table 1: Table of primers sequence.**

| Primer | Assession no. | Base pair sequence |
| --- | --- | --- |
| CGRP Fw | NM_001033954 | CTGGTTGTCAGCATCTTG |
| CGRP Rv |  | GGCGAACTTCTTCTTCAC |
| SP Fw | NM_009311 | AGCACAGTGATGAAGGAG |
| SP Rv |  | GGATACAAATAGAGTCAAATACC |
| GAP-43 Fw | NM_008083.2 | AGCCTAAACAAGCCGATGTG |
| GAP-43 Rv |  | GCAGGAGAGACAGGGTTCAG |
| Ntrk1 Fw | NM_001033124.1 | TCTCGCCAGTGGACGGTAA |
| Ntrk1 Rv |  | AATACAGCAGGGCGGTTGAT |
| RhoA Fw | NM_016802.4 | TGTGTTTTTCCATTGACAGCCC |
| RhoA Rv |  | TGTTCCCAACCAGGATGATGG |
| Gskip Fw | NM_178613.3 | AGCTCACAGAAGCAGGGCTC |
| Gskip Rv |  | AGGCAGGGCTGAGAGTATCC |
